# Association between COVID‐19 and sensorineural hearing loss: Evidence from a Mendelian randomization study in European and East Asian population

**DOI:** 10.1002/iid3.1108

**Published:** 2023-12-06

**Authors:** Fengyang Wang, Qiuyuan Yin, Lei Zhu

**Affiliations:** ^1^ Henan Provincial Institute of Medical Genetics, People's Hospital of Zhengzhou University Henan Provincial People's Hospital Zhengzhou China; ^2^ State Key Laboratory for Conservation and Utilization of Bio‐Resources in Yunnan, School of Life Sciences Yunnan University Kunming China

**Keywords:** causal effect, long COVID‐19, Mendelian randomization study, severe acute respiratory syndrome coronavirus 2, sensorineural hearing loss

## Abstract

**Background:**

Long coronavirus disease (COVID), characterized by persistent and sometimes debilitating symptoms following a severe acute respiratory syndrome coronavirus 2 (SARS‐CoV‐2) infection, has garnered increasing attention as a potential public health crisis. Emerging evidence indicates a higher incidence of hearing loss in individuals who have had COVID 2019 (COVID‐19) compared to the general population. However, the conclusions were inconsistent, and the causal relationship between COVID‐19 and sensorineural hearing loss remains unknown.

**Methods:**

To addresses this outstanding issue, we performed Mendelian randomization analysis to detect the causal association between COVID‐19 and hearing loss using the largest genome‐wide association study data to date in the European population and confirmed the results in the East Asian population. Comprehensively sensitive analyses were followed, including Cochran's *Q* test, Mendelian randomization (MR)‐Egger intercept test, MR‐pleiotropy residual sum and outlier, and leave‐one‐out analysis, to validate the robustness of our results.

**Results:**

Our results suggested that there is no causal association between COVID‐19 and the risk of hearing loss in the European population. Neither the susceptibility, hospitalization, and severity of COVID‐19 on hearing loss (inverse variance weighted method: odds ratio (OR) = 1.046, 95% confidence interval (CI) = 0.907–1.205, *p* = .537; OR = 0.995, 95% CI = 0.956–1.036, *p* = .823; OR = 0.995, 95% CI = 0.967–1.025, *p* = .76). Replicated analyses in the East Asian population yielded consistent results. No pleiotropy and heterogeneity were found in our results.

**Conclusion:**

In conclusion, our MR results do not support a genetically predicted causal relationship between COVID‐19 and sensorineural hearing loss. Thus, the associations observed in prior observational studies may have been influenced by confounding factors rather than a direct cause‐and‐effect relationship. More clinical and mechanism research are needed to further understand this association in the future.

## 1 INTRODUCTION

Over the past 3 years, coronavirus disease 2019 (COVID‐19), which is caused by the severe acute respiratory syndrome coronavirus 2 (SARS‐CoV‐2), has indeed become a global pandemic. This viral outbreak has spread across the world, leading to significant mortality and morbidity on a global scale.^1^ The COVID‐19 pandemic is still developing at the time of writing, with more than 770 million illnesses and nearly 7 million fatalities documented (https://covid19.who.int/).^1^ According to conservative projections, more than 200 million SARS‐CoV‐2 infected people would experience COVID‐19 symptoms that last for a long time, commonly known as “long COVID” or “post‐COVID conditions.” It has already debilitated millions of individuals worldwide, and that number is continuing to grow.^2^ Long COVID has been considered as the next public health crisis, however its symptoms remain largely unexplained,^3^ and the causal relationship between COVID‐19 and its symptoms is even less well understood, such as hearing loss.

Hearing loss seriously interferes with the quality of life, however hearing loss following COVID‐19 infection has been controversial for years. Previous studies about the incidence of sensorineural hearing loss during the COVID‐19 pandemic had conflicting results.^4^, ^5^, ^6^ In addition, some meta‐analyses and observational studies confirmed that COVID‐19 can cause hearing loss^7^, ^8^ and some cases of hearing loss following SARS‐CoV‐2 infection have also been reported among children,^9^ but other studies showed that the effect of COVID‐19 on auditory symptoms was negligible.^10^, ^11^, ^12^, ^13^ Traditional epidemiological conclusions may be disturbed by confounding interference and reverse causation.

Mendelian randomization (MR) is a research approach that uses genetic variants as natural experiments to assess potential causal relationships between an exposure (e.g., a risk factor) and an outcome (e.g., a disease).^14^ Due to the random assignment of a person's genetic variations at birth, confounding bias can be reduced in MR studies. Similar to the last example, reverse causation also can be prevented since genetic variations are assigned before a disease manifests itself. MR has been widely applied to explore the causal association between COVID‐19 and its symptoms, including autoimmune diseases,^15^ aging,^16^ metabolic diseases,^17^ and cardiovascular comorbidities.^18^ In this work, we used MR methods to reveal the causal relationship between COVID‐19 and sensorineural hearing loss.

## 2 METHODS

### 2.1 Study design

We gathered our summary‐level genome‐wide association studies (GWAS) data from published research, all of whose data had been authorized by institutional review boards. A two‐sample MR method was conducted to detect the causal association between hearing loss and three types of COVID‐19 infection.

### 2.2 Data sources

We performed MR studies based on summary statistics of GWAS in the European population and validated the results in the East Asian population. Summary statistics for COVID‐19‐related traits in both European and East Asian populations were sourced from the latest release of the COVID‐19 Host Genetics Initiative GWAS meta‐analyses. These summary statistics encompass three distinct sets of genetic instruments, specifically focused on SARS‐CoV‐2 infection, hospitalized COVID‐19 cases, and severe COVID‐19 cases. GWAS summary statistics for sensorineural hearing loss in European population were obtained from the FinnGen consortium R9 release data (32,487 cases and 331,736 controls).^19^ GWAS summary statistics for hearing loss in East Asian population were obtained from the BioBank Japan (3400 cases and 175,326 controls).^20^ Detailed information for the data sources was presented in Table S1.

### 2.3 Selection criteria of instrumental variables (IVs)

Similar to most MR studies, a genome‐wide significance threshold of *p* < 5 × 10^−8^ was employed to screen single nucleotide polymorphisms (SNPs) in European population. Because of a limited number of SNPs meeting genome‐wide significance in East Asian population, we used SNPs with a more relaxed threshold (*p* < 5 × 10^−6^) as potential IVs of COVID‐19 in East Asian population. To ensure the independence of IVs in our analysis, we implemented a linkage disequilibrium (LD) clumping procedure. Specifically, we utilized a clumping window of 10 MB and set a stringent LD threshold of *R* ^2^ < 0.001 based on European ancestry reference data from the 1000 Genomes Project. Meanwhile, to avoid bias owing to the employment of weak instruments, F statistics were calculated for each SNP to measure the statistical strength, and only strong IVs (F‐statistics >10) for each of our exposure of interest remained. Ambiguous and palindromic SNPs of which the effect cannot be correct in the harmonizing process were excluded. To mitigate the risk of false positives in MR analysis, we excluded SNPs that are directly associated with the outcome.

We also scanned with the PhenoScanner V2 (www.phenoscanner.medschl.cam.ac.uk), a database of human genotype‐phenotype associations, to detect whether these IVs were associated with the potential risk factors of outcome, including hypertension, smoking, diabetes, and viral infection,^21^ and SNPs associated with any of these potential confounders were removed.

### 2.4 Statistical analyses

Random‐effect inverse‐variance weighted (IVW) method was used as the main analysis, which combined the Wald ratio of each SNP on the outcome and obtained a pooled causal estimate, to verify the causal relationship between COVID‐19 and hearing loss. If horizontal pleiotropy was not existed, the IVW method would be impartial.^22^ Another two MR methods, MR‐Egger and weighted median, were conducted to ensure the robustness of the results. All genetic variations were allowed to have pleiotropic effects under MR‐Egger method, but such effects must be unrelated to the variant‐exposure connection.^23^ The weighted median method could give a correct estimation of causal association when up to half of IVs are invalid.^24^ The pleiotropy was calculated using the intercept from MR‐Egger regression,^25^ and heterogeneity was determined using Cochran's Q test in the IVW approach.^26^ MR‐Pleiotropy Residual Sum and Outlier methods (MR‐PRESSO) were also used to detect and correct horizontal pleiotropy.^27^ We also identified whether existed any high‐influence SNPs biasing the pooled effect estimates by leave‐one‐out analysis. In this study, we considered three clinical types of COVID‐19 infection. To address multiple comparisons, we applied the Bonferroni correction by dividing the conventional *p* value threshold of .05 by 3 (the number of tests), resulting in a significance threshold of *p* < .017.

## 3 RESULTS

### 3.1 IV selection

We performed MR studies based on summary statistics of GWAS in the European population and validated the results in the East Asian population. Detailed information for the data sources was presented in Table S1. To avoid potential pleiotropic, SNPs related to potential risk factors of outcome (e.g. hearing loss, hypertension, smoking, diabetes, autoimmune disorders and viral infection) were removed employing PhenoScanner searches (http://www. phenoscanner. medschl. cam. ac. uk/),^28^ as presented in Table S2. To avoid bias owing to the employment of weak instruments, the *F*‐statistic of each SNP was calculated and removed the SNP with *F*‐statistic <10. After removing potential confounders associated with outcome and weak genetic instruments, ultimately we got 10 IVs for SARS‐CoV‐2 infection, 28 IVs for hospitalized COVID‐19, and 22 IVs for severe COVID‐19, were included in the MR analysis of European population. The used genetic instruments of exposures in European population were shown in Table S3.

### 3.2 Causal relationship between COVID‐19 and hearing loss in european population

**Figure 1:**
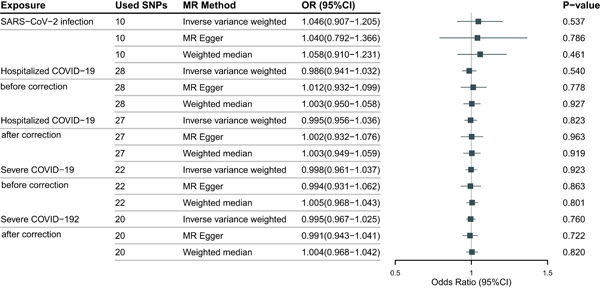
The causal effect of COVID‐19 on the risk of hearing loss in European population.

The results suggested that there is no causal association between COVID‐19 and the risk of hearing loss. Neither the susceptibility, hospitalization, and severity of COVID‐19 on hearing loss (IVW method: odds ratio (OR) = 1.046, 95% confidence interval (CI) = 0.907–1.205, *p* = .537; OR = 0.986, 95% CI = 0.941–1.032, *p* = .54; OR = 0.998, 95% CI = 0.961–1.037, *p* = .923). The results from MR‐Egger and weighted median were consistent with IVW results (Figure 1). No pleiotropy was found in the sensitivity test. However, heterogeneity was observed with a Cochran *Q*‐test in Hospitalized COVID‐19 and Severe COVID‐19 exposure (Table S4). Then we used the MR‐PRESSO to detect the outliers SNPs, and one SNP (rs1634761) for Hospitalized COVID‐19 and two SNPs (rs2236645, rs343320) for Severe COVID‐19 were removed (Table S5). The MR approaches were re‐applied to evaluate the relationship between COVID‐19 and hearing loss and the results still do not support the causal association from the hospitalization or severity of COVID‐19 on hearing loss (Figure 1) (IVW method: OR = 0.995, 95% CI = 0.956–1.036, *p* = .823; OR = 0.995, 95% CI = 0.967–1.025, *p* = .76). Leave‐one‐out sensitivity analysis showed that no SNPs independently drove the results, indicating the reliability of our results (Table S6).

### 3.3 Causal relationship between COVID‐19 and hearing loss in East Asian population

**Figure 2:**
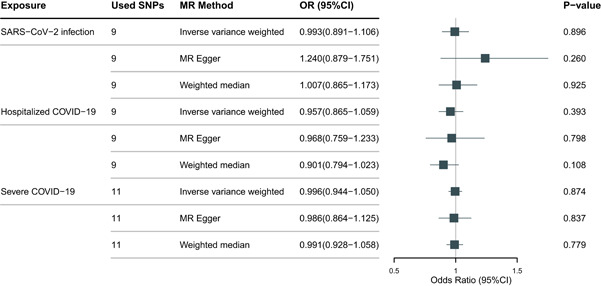
The causal effect of COVID‐19 on the risk of hearing loss in East Asian population.

To make our results more robust, we also detect the causal relationship between COVID‐19 and hearing loss in East Asian population. The IVs of COVID‐19 in East Asian population were selected by the same criteria of European population. The used IVs of exposures in East Asian population were shown in Table S7. Validation analysis in the East Asian population showed the same results that susceptibility, hospitalization, and severity of COVID‐19 were not causally associated with the risk of hearing loss (Figure 2), and no pleiotropy and heterogeneity were found (Tables S8, S9, S10).

## 4 DISCUSSION

In our study, we investigated the potential causal relationship between COVID‐19 and hearing loss using three complementary MR methods. Our MR analysis did not provide strong evidence to support a causal association between COVID‐19 and the risk of hearing loss in both European and East Asian populations. These findings suggest that other factors or mechanisms may contribute to hearing loss in individuals affected by COVID‐19, and further research is needed to fully understand this relationship.

Viral infections are known to cause hearing loss, ranging from mild to profound, and can affect one or both ears. This hearing loss can be conductive or sensorineural in nature.^29^ Hearing loss resulting from viral infections can be attributed to factors such as inflammation of the auditory pathway and cross‐reactions between antigens in the inner ear and the virus.^30^ The impact of hearing loss varies considerably depending on the specific virus involved, highlighting the diverse effects that different viruses can have on auditory health.^29^ Previous studies have suggested that the novel coronavirus is neurotrophic and neuroinvasive,^31^ which means it's likely to affect the hearing system. Some studies suggested SARS‐CoV‐2 infection as a precursor of hearing loss^32^ and considered hearing loss as one of the clinical manifestations of SARS‐CoV‐2 infection, especially sensorineural hearing loss.^33^ Several theories and hypotheses have been proposed to explain the mechanisms underlying hearing loss following COVID‐19 infection. For instance, activation of the immune system and inflammatory responses may have adverse effects on the auditory system; SARS‐CoV‐2 infection causes the deoxygenation of erythrocytes which raises the possibility of hypoxia in the auditory center, leading to persistent hearing loss; COVID‐19 could affect blood circulation and leads to ischemia, which might damage the auditory system.^33^, ^34^, ^35^ However, a meta‐analysis based on 350 studies showed a low pooled prevalence (3%) of hearing impairment in COVID‐19 patients, which implied the limited effects of COVID‐19 infection on hearing loss.^36^ In addition, some observational studies showed that there were no statistically significant differences in frequencies of hearing loss between the COVID‐19 positive patients and the control group.^10^, ^11^, ^12^, ^13^

The inconsistent findings in previous observational studies examining the association between COVID‐19 and hearing loss could be attributed to several factors: First, the sample size is too small, which affect the accuracy of the results.^37^ The low prevalence of hearing impairment in COVID‐19 patients make it difficult to collect enough samples.^36^ Second, many latent confounders will affect the results of previous observational studies.^38^ Such as, treatment options and medical conditions for COVID‐19, age of patients, environment, dietary patterns, and lifestyle. Third, the effects of COVID‐19 on hearing loss may function through some shared pathways, not COVID‐19 itself.

This study had several advantages. First, MR analysis was considered as natural randomized controlled trials which are widely accepted in causal research. Compared to the observational study, using MR design, our study is largely free from reverse causation and residual confounding. Besides, GWAS summary data used in this study were obtained from the largest scale of meta‐studies to date, ensuring the strength of instruments in the MR analysis. Third, we verified the causal association between COVID‐19 and hearing loss in two population, which make results more robust.

However, this study also had some limitations and should be noted while interpreting the results. First, since the limited number of SNPs reached genome‐wide significance (*p* < 5 × 10^−8^), we thus relaxed the *P* threshold in East Asian population. Second, there may be participants who are included in both the exposure (COVID‐19) and outcome (hearing loss) groups, but it was difficult to accurately quantify the proportions of these participants. Third, there are different subtypes of sensorineural hearing loss, such as presbycusis, noise‐induced hearing loss, sudden sensorineural hearing loss and others. Detailed subgroup analyses were unable to be performed since summary statistics rather than raw data were used in the analysis.

Long COVID posts a global health challenge for the society. To deal with long COVID, it is important to understand the relationship between COVID‐19 and its symptoms. Previous conflicting evidence on the relationship between COVID‐19 and hearing loss may mislead the treatment strategies for long COVID‐19. Our findings help to understand COVID‐19 on hearing loss and provide valuable suggestions for the diagnosis and treatment of long COVID‐19.

## 5 CONCLUSION

In conclusion, our MR results do not support the causal association between COVID‐19 and sensorineural hearing loss. Our findings help to understand COVID‐19 on sensorineural hearing loss and provide valuable suggestions for the diagnosis and treatment of long COVID‐19. More clinical and mechanism research are needed to further understand this association in the future.

## AUTHOR CONTRIBUTIONS

**Fengyang Wang**: Investigation; methodology. **Qiuyuan Yin**: Data curation; funding acquisition; investigation; methodology.

## CONFLICT OF INTEREST STATEMENT

The authors declare no conflict of interest.

## ETHICS STATEMENT

This research has been conducted using published studies and consortia providing publicly available summary statistics. All original studies have been approved by the corresponding ethical review board, and the participants have provided informed consent. In addition, no individual‐level data were used in this study. Therefore, no new ethical review board approval was required.

## Supporting information

Supporting information.

## Data Availability

All data are publicly available. Details see Table S1.
