# Supplementary material for: Association between COVID‐19 and sensorineural hearing loss: Evidence from a Mendelian randomization study in European and East Asian population
Source: Immun Inflamm Dis. 2023 Dec 6;11(12):e1108. doi: 10.1002/iid3.1108 (PMC10698807; doi:10.1002/iid3.1108)
Supplement: Supplementary file 1 — Supporting information. [file IID3-11-e1108-s001.docx]

**Supplementary Table 1.** Detailed information on GWAS summary data used in this study.

| **Phenotype** | **Sexes** | **Release version** | **Consortium** | **Sample size** | **Cases** | **Controls** | **Population** | **PMID** |
| --- | --- | --- | --- | --- | --- | --- | --- | --- |
| **European population** | | | | | | | | |
| SARS-CoV-2 infection | combined | R7 | COVID-19 Host Genetics Initiative | 2,297,856 | 122,616 | 2,475,240 | European | NA |
| Hospitalized COVID-19 | combined | R7 | COVID-19 Host Genetics Initiative | 2,095,324 | 32,519 | 2,062,805 | European | NA |
| Severe COVID-19 | combined | R7 | COVID-19 Host Genetics Initiative | 1,086,211 | 13,769 | 1,072,442 | European | NA |
| Hearing Loss | combined | R9 | FinnGen | 364,223 | 32,487 | 331,736 | European | NA |
| **East Asian population (Validation set)** | | | | | | | | |
| SARS-CoV-2 infection | combined | R7 | COVID-19 Host Genetics Initiative | 40,580 | 4,459 | 36,121 | East Asian | NA |
| Hospitalized COVID-19 | combined | R7 | COVID-19 Host Genetics Initiative | 34,082 | 2,882 | 31,200 | East Asian | NA |
| Severe COVID-19 | combined | R7 | COVID-19 Host Genetics Initiative | 5,656 | 794 | 4,862 | East Asian | NA |
| Hearing Loss | combined | Sakaue et al., 2021 | BioBank Japan | 178,726 | 3,400 | 175,326 | East Asian | 34594039 |

Source of COVID-19 data: https://www.covid19hg.org/results/r7/

Source of FinnGen hearing loss data: https:// r9.finngen.fi/pheno/H8_HL_SEN_NAS

Source of BioBank Japan hearing loss data: https:// pheweb.jp/pheno/Hearing_Loss

**Supplementary Table 2.** Excluded SNPs of Potential confounders of hearing loss (European population).

| Filter through PhenoScanner database | | |
| --- | --- | --- |
| Exposures | **Exclude SNPs** | **Traits** |
| SARS-CoV-2 infection^a^ | rs11264339 | Past tobacco smoking, Smoking status: previous, |
|  | rs9264740 | Diabetes diagnosed by doctor, Medication for cholesterol, blood pressure or diabetes: insulin, Self-reported type 1 diabetes, Treatment with insulin, |
| Hospitalized COVID-19^a^ | rs63750417 | Hearing difficulty or problems with background noise |
|  | rs492602 | Self-reported hypertension, |
| Severe COVID-19^a^ | rs62056905 | Hearing difficulty or problems with background noise |
|  | rs1128175 | HIV 1 control, Diabetes diagnosed by doctor, Started insulin within one year diagnosis of diabetes, Medication for cholesterol, blood pressure or diabetes |

a: SNPs associated with potential confounders of hearing loss, i.e. hearing difficulty, hypertension, smoking, diabetes, viral infection, were removed. Potential confounders for hearing loss were obtained from World report on hearing 2021 from World Health Organization, ISBN 978-92-4-002049-8.

Potential confounders SNPs were filtered under the condition of P < 1×10^-5^ in the PhenoScanner database. (PhenoScanner database: http://www.phenoscanner.medschl.cam.ac.uk)

**Supplementary Table 3.** Detailed information on genetic instruments of exposures after removing potential confounders associated with hearing loss(European population).

| Exposure | SNP | Effect_allele | Other_allele | EAF | BETA | SE | *P*-value | F-statistic^a^ |
| --- | --- | --- | --- | --- | --- | --- | --- | --- |
| SARS-CoV-2 infection | rs10774673 | T | C | 0.675 | 0.029 | 0.005 | 2.14E-09 | 35.8 |
| SARS-CoV-2 infection | rs1123573 | G | A | 0.379 | -0.026 | 0.005 | 3.16E-08 | 30.6 |
| SARS-CoV-2 infection | rs12610495 | G | A | 0.303 | 0.05 | 0.005 | 1.80E-22 | 95.1 |
| SARS-CoV-2 infection | rs2260685 | C | T | 0.474 | 0.033 | 0.005 | 1.01E-12 | 50.8 |
| SARS-CoV-2 infection | rs2290859 | T | C | 0.351 | -0.051 | 0.005 | 1.32E-25 | 109.4 |
| SARS-CoV-2 infection | rs2834158 | C | T | 0.661 | -0.041 | 0.005 | 8.51E-17 | 69.3 |
| SARS-CoV-2 infection | rs35044562 | G | A | 0.078 | 0.128 | 0.008 | 1.64E-51 | 228 |
| SARS-CoV-2 infection | rs676314 | G | A | 0.326 | 0.028 | 0.005 | 1.02E-08 | 32.8 |
| SARS-CoV-2 infection | rs7118388 | G | A | 0.506 | 0.027 | 0.005 | 4.71E-09 | 34.3 |
| SARS-CoV-2 infection | rs73062389 | A | G | 0.054 | 0.2 | 0.01 | 7.61E-92 | 413.1 |
| Hospitalized COVID-19 | rs1123573 | G | A | 0.377 | -0.069 | 0.01 | 4.13E-11 | 43.6 |
| Hospitalized COVID-19 | rs117169628 | A | G | 0.144 | 0.101 | 0.014 | 1.27E-13 | 54.9 |
| Hospitalized COVID-19 | rs12610495 | G | A | 0.304 | 0.148 | 0.011 | 4.21E-41 | 180.3 |
| Hospitalized COVID-19 | rs139589338 | G | A | 0.019 | 0.209 | 0.036 | 4.97E-09 | 34.2 |
| Hospitalized COVID-19 | rs149533170 | A | G | 0.008 | 0.284 | 0.051 | 2.78E-08 | 30.9 |
| Hospitalized COVID-19 | rs1498399 | G | A | 0.386 | 0.069 | 0.01 | 8.24E-12 | 46.7 |
| Hospitalized COVID-19 | rs1634761 | T | C | 0.494 | -0.068 | 0.009 | 1.02E-12 | 50.8 |
| Hospitalized COVID-19 | rs17279437 | A | G | 0.107 | -0.114 | 0.017 | 1.37E-11 | 45.7 |
| Hospitalized COVID-19 | rs17412601 | C | T | 0.351 | -0.068 | 0.01 | 3.69E-11 | 43.8 |
| Hospitalized COVID-19 | rs2102497 | C | T | 0.734 | 0.064 | 0.012 | 4.21E-08 | 30 |
| Hospitalized COVID-19 | rs2326562 | T | C | 0.381 | 0.053 | 0.01 | 4.81E-08 | 29.8 |
| Hospitalized COVID-19 | rs2897075 | T | C | 0.374 | 0.059 | 0.01 | 2.88E-09 | 35.3 |
| Hospitalized COVID-19 | rs3014983 | T | C | 0.736 | -0.07 | 0.012 | 6.99E-09 | 33.5 |
| Hospitalized COVID-19 | rs34712979 | A | G | 0.25 | -0.064 | 0.011 | 2.17E-08 | 31.3 |
| Hospitalized COVID-19 | rs35705950 | T | G | 0.107 | -0.099 | 0.016 | 1.90E-10 | 40.6 |
| Hospitalized COVID-19 | rs41264915 | G | A | 0.097 | -0.143 | 0.015 | 1.43E-20 | 86.5 |
| Hospitalized COVID-19 | rs41435745 | C | G | 0.029 | 0.213 | 0.035 | 1.45E-09 | 36.6 |
| Hospitalized COVID-19 | rs4475253 | G | A | 0.324 | 0.058 | 0.01 | 7.07E-09 | 33.5 |
| Hospitalized COVID-19 | rs45524632 | A | C | 0.019 | 0.223 | 0.035 | 1.07E-10 | 41.7 |
| Hospitalized COVID-19 | rs4767025 | T | C | 0.676 | 0.075 | 0.01 | 7.13E-14 | 56 |
| Hospitalized COVID-19 | rs5023077 | C | T | 0.491 | -0.066 | 0.01 | 3.79E-12 | 48.2 |
| Hospitalized COVID-19 | rs61078946 | T | A | 0.111 | -0.09 | 0.016 | 3.12E-08 | 30.6 |
| Hospitalized COVID-19 | rs61882275 | A | G | 0.342 | -0.092 | 0.01 | 2.30E-20 | 85.5 |
| Hospitalized COVID-19 | rs676314 | G | A | 0.325 | 0.078 | 0.01 | 8.95E-15 | 60.1 |
| Hospitalized COVID-19 | rs67959919 | A | G | 0.077 | 0.492 | 0.018 | 2.36E-173 | 787.9 |
| Hospitalized COVID-19 | rs78295726 | T | C | 0.154 | 0.074 | 0.013 | 1.42E-08 | 32.2 |
| Hospitalized COVID-19 | rs78314212 | T | C | 0.085 | 0.122 | 0.017 | 1.22E-12 | 50.5 |
| Hospitalized COVID-19 | rs9636867 | G | A | 0.336 | 0.128 | 0.01 | 2.05E-36 | 158.8 |
| Severe COVID-19 | rs10066378 | C | T | 0.116 | 0.118 | 0.021 | 1.96E-08 | 31.5 |
| Severe COVID-19 | rs10850097 | T | C | 0.671 | 0.095 | 0.015 | 1.71E-10 | 40.8 |
| Severe COVID-19 | rs11208559 | G | C | 0.291 | 0.103 | 0.017 | 2.17E-09 | 35.8 |
| Severe COVID-19 | rs1123573 | G | A | 0.37 | -0.106 | 0.015 | 2.80E-12 | 48.8 |
| Severe COVID-19 | rs11614702 | A | G | 0.507 | 0.101 | 0.014 | 2.33E-13 | 53.7 |
| Severe COVID-19 | rs117169628 | A | G | 0.137 | 0.157 | 0.02 | 4.36E-15 | 61.5 |
| Severe COVID-19 | rs12534422 | T | C | 0.303 | 0.086 | 0.015 | 1.34E-08 | 32.3 |
| Severe COVID-19 | rs12610495 | G | A | 0.313 | 0.242 | 0.016 | 3.05E-51 | 226.8 |
| Severe COVID-19 | rs12614007 | A | G | 0.749 | 0.094 | 0.017 | 2.46E-08 | 31.1 |
| Severe COVID-19 | rs17279437 | A | G | 0.1 | -0.172 | 0.025 | 7.29E-12 | 46.9 |
| Severe COVID-19 | rs17713054 | A | G | 0.075 | 0.756 | 0.026 | 1.09E-185 | 844.6 |
| Severe COVID-19 | rs2236645 | T | C | 0.085 | 0.179 | 0.025 | 6.46E-13 | 51.7 |
| Severe COVID-19 | rs28368148 | G | C | 0.023 | 0.449 | 0.065 | 7.26E-12 | 47 |
| Severe COVID-19 | rs2897075 | T | C | 0.376 | 0.088 | 0.014 | 9.32E-10 | 37.5 |
| Severe COVID-19 | rs343320 | A | G | 0.07 | 0.154 | 0.028 | 2.06E-08 | 31.4 |
| Severe COVID-19 | rs34712979 | A | G | 0.254 | -0.11 | 0.017 | 1.21E-10 | 41.4 |
| Severe COVID-19 | rs35705950 | T | G | 0.108 | -0.164 | 0.023 | 6.95E-13 | 51.6 |
| Severe COVID-19 | rs41264915 | G | A | 0.094 | -0.206 | 0.023 | 2.09E-19 | 81.2 |
| Severe COVID-19 | rs568035 | T | C | 0.063 | 0.143 | 0.026 | 3.60E-08 | 30.4 |
| Severe COVID-19 | rs60132559 | T | C | 0.32 | 0.091 | 0.015 | 1.11E-09 | 37.1 |
| Severe COVID-19 | rs61882275 | A | G | 0.343 | -0.126 | 0.015 | 8.08E-18 | 73.9 |
| Severe COVID-19 | rs9636867 | G | A | 0.331 | 0.184 | 0.015 | 3.47E-34 | 148.6 |

EAF, effect allele frequency;

The genetic instrumental variables were filtered with genome‐wide significance through *P<5e-08*, and removed Linkage disequilibrium (LD) using a clumping r2 cutoff of 0.001 within a 10 Mb window, using the 1000 Genomes Project Phase 3 (EUR) as the reference panel.

a: the F-statistic for each SNP was calculated as follows: F=(BATA/SE)^2

**Supplementary Table 4.** The results of pleiotropy test, Cochrane’s Q between COVID-19 and hearing loss (European population)

| Exposure&Outcomes | Egger_intercept | *P* for pleiotropy^a^ | Cochrane’s Q | *P* for Cochrane’s Q^b^ |
| --- | --- | --- | --- | --- |
| SARS-CoV-2 infection | 0.00034 | 0.96 | 12.17 | 0.2 |
| Hospitalized COVID-19 | -0.0035 | 0.45 | 42.63 | 0.03 |
| Severe COVID-19 | 0.00087 | 0.88 | 43.55 | <0.01 |

a: P-values for pleiotropy were derived from MR-Egger test and *P*-value<0.05 indicates a possible pleiotropic effect.

b: P-values for Cochrane’s Q were derived from Cochrane’s Q test and *P*-value<0.05 indicates a possible heterogeneity.

**Supplementary Table 5.** MR-PRESSO analysis for the association between COVID-19 and hearing loss(European population).

| **Exposure** | **MR Analysis** | **Causal Estimate** | **Sd** | **T-stat** | **P-value** | **RSS_obs_** | **globalPvalue** | **outlier SNP** |
| --- | --- | --- | --- | --- | --- | --- | --- | --- |
| SARS-CoV-2 infection | RAW | 0.0447 | 0.0725 | 0.6171 | 0.5524 | 13.84 | 0.266 | 0 |
|  | Outlier-corrected | NA | NA | NA | NA | NA | NA |  |
| Hospitalized COVID-19 | RAW | -0.0144 | 0.0235 | -0.6124 | 0.5454 | 45.06 | 0.038 | rs1634761 |
|  | Outlier-corrected | -0.0046 | 0.0205 | -0.2238 | 0.8247 | 31.88 | 0.292 |  |
| Severe COVID-19 | RAW | -0.0019 | 0.0194 | -0.0970 | 0.9236 | 45.74 | 0.006 | rs2236645, rs343320 |
|  | Outlier-corrected | -0.0045 | 0.0148 | -0.3061 | 0.7629 | 23.57 | 0.346 |  |

**Supplementary Table 6.** The leave-one-out analysis for COVID-19 on hearing loss (European population)

| Exposure | SNP | BETA | SE | *P*-value |
| --- | --- | --- | --- | --- |
| SARS-CoV-2 infection | rs10774673 | 0.024 | 0.068 | 0.723 |
|  | rs1123573 | 0.0639 | 0.069 | 0.351 |
|  | rs12610495 | 0.0348 | 0.081 | 0.668 |
|  | rs2260685 | 0.0188 | 0.07 | 0.787 |
|  | rs2290859 | 0.0713 | 0.078 | 0.36 |
|  | rs2834158 | 0.0725 | 0.072 | 0.317 |
|  | rs35044562 | 0.0504 | 0.089 | 0.571 |
|  | rs676314 | 0.0375 | 0.077 | 0.627 |
|  | rs7118388 | 0.0462 | 0.078 | 0.555 |
|  | rs73062389 | 0.0284 | 0.086 | 0.742 |
|  | **All** | **0.0447** | **0.072** | **0.537** |
| Hospitalized COVID-19 | rs1123573 | -0.0106 | 0.024 | 0.654 |
|  | rs117169628 | -0.0089 | 0.023 | 0.7 |
|  | rs12610495 | -0.0201 | 0.025 | 0.421 |
|  | rs139589338 | -0.0146 | 0.024 | 0.546 |
|  | rs149533170 | -0.0132 | 0.024 | 0.577 |
|  | rs1498399 | -0.0124 | 0.024 | 0.607 |
|  | rs1634761 | -0.0046 | 0.02 | 0.823 |
|  | rs17279437 | -0.0123 | 0.024 | 0.61 |
|  | rs17412601 | -0.0127 | 0.024 | 0.599 |
|  | rs2102497 | -0.0166 | 0.024 | 0.489 |
|  | rs2326562 | -0.018 | 0.023 | 0.441 |
|  | rs2897075 | -0.0132 | 0.024 | 0.584 |
|  | rs3014983 | -0.015 | 0.024 | 0.536 |
|  | rs34712979 | -0.0118 | 0.024 | 0.62 |
|  | rs35705950 | -0.0158 | 0.024 | 0.513 |
|  | rs41264915 | -0.0122 | 0.024 | 0.611 |
|  | rs41435745 | -0.0145 | 0.025 | 0.556 |
|  | rs4475253 | -0.0126 | 0.024 | 0.6 |
|  | rs45524632 | -0.0166 | 0.024 | 0.486 |
|  | rs4767025 | -0.0199 | 0.023 | 0.388 |
|  | rs5023077 | -0.0167 | 0.024 | 0.487 |
|  | rs61078946 | -0.0135 | 0.024 | 0.575 |
|  | rs61882275 | -0.0158 | 0.024 | 0.515 |
|  | rs676314 | -0.017 | 0.024 | 0.479 |
|  | rs67959919 | -0.0236 | 0.029 | 0.422 |
|  | rs78295726 | -0.0149 | 0.024 | 0.536 |
|  | rs78314212 | -0.0214 | 0.022 | 0.338 |
|  | rs9636867 | -0.0083 | 0.025 | 0.736 |
|  | **All** | **-0.0144** | **0.024** | **0.54** |
| Severe COVID-19 | rs10066378 | -0.0021 | 0.02 | 0.915 |
|  | rs10850097 | -0.0053 | 0.019 | 0.783 |
|  | rs11208559 | -0.0018 | 0.02 | 0.929 |
|  | rs1123573 | 0.0014 | 0.02 | 0.944 |
|  | rs11614702 | -0.0045 | 0.02 | 0.821 |
|  | rs117169628 | 0.0028 | 0.019 | 0.883 |
|  | rs12534422 | -0.0035 | 0.02 | 0.86 |
|  | rs12610495 | -0.0058 | 0.021 | 0.783 |
|  | rs12614007 | -0.0023 | 0.02 | 0.907 |
|  | rs17279437 | 0 | 0.02 | 1 |
|  | rs17713054 | -0.0049 | 0.026 | 0.85 |
|  | rs2236645 | -0.0093 | 0.018 | 0.598 |
|  | rs28368148 | -9.00E-04 | 0.02 | 0.964 |
|  | rs2897075 | -8.00E-04 | 0.02 | 0.968 |
|  | rs343320 | 0.0028 | 0.017 | 0.872 |
|  | rs34712979 | 6.00E-04 | 0.02 | 0.976 |
|  | rs35705950 | -0.0029 | 0.02 | 0.886 |
|  | rs41264915 | -1.00E-04 | 0.02 | 0.997 |
|  | rs568035 | -0.0037 | 0.02 | 0.851 |
|  | rs60132559 | -0.0033 | 0.02 | 0.867 |
|  | rs61882275 | -0.0026 | 0.02 | 0.897 |
|  | rs9636867 | 0.0034 | 0.02 | 0.867 |
|  | **All** | **-0.0019** | **0.019** | **0.923** |

**Supplementary Table 7.** Detailed information on genetic instruments of exposures (East Asian population)

| Exposure | SNP | Effect_allele | Other_allele | EAF | BETA | SE | *P*-value | F-statistic^a^ |
| --- | --- | --- | --- | --- | --- | --- | --- | --- |
| SARS-CoV-2 infection | rs10181451 | T | G | 0.056 | -0.287 | 0.059 | 1.04E-06 | 23.9 |
| SARS-CoV-2 infection | rs12246187 | G | C | 0.145 | -0.204 | 0.042 | 1.06E-06 | 23.8 |
| SARS-CoV-2 infection | rs12425722 | C | T | 0.048 | -0.298 | 0.061 | 1.00E-06 | 23.9 |
| SARS-CoV-2 infection | rs12540473 | A | G | 0.045 | -0.296 | 0.064 | 3.20E-06 | 21.7 |
| SARS-CoV-2 infection | rs12960174 | T | C | 0.023 | -0.424 | 0.092 | 3.75E-06 | 21.4 |
| SARS-CoV-2 infection | rs183690023 | A | G | 0.017 | 0.642 | 0.132 | 1.08E-06 | 23.8 |
| SARS-CoV-2 infection | rs315664 | C | T | 0.725 | -0.167 | 0.034 | 7.30E-07 | 24.5 |
| SARS-CoV-2 infection | rs76497367 | A | C | 0.055 | 0.326 | 0.071 | 3.79E-06 | 21.4 |
| SARS-CoV-2 infection | rs9367106 | C | G | 0.312 | 0.197 | 0.033 | 2.53E-09 | 35.5 |
| Hospitalized COVID-19 | rs11066150 | A | G | 0.372 | -0.167 | 0.035 | 2.20E-06 | 22.4 |
| Hospitalized COVID-19 | rs111553899 | A | G | 0.052 | -0.421 | 0.086 | 1.03E-06 | 23.9 |
| Hospitalized COVID-19 | rs11919142 | A | G | 0.041 | 0.369 | 0.079 | 3.45E-06 | 21.5 |
| Hospitalized COVID-19 | rs12175265 | A | G | 0.31 | 0.273 | 0.038 | 3.73E-13 | 52.8 |
| Hospitalized COVID-19 | rs144808322 | G | T | 0.008 | 0.98 | 0.21 | 3.15E-06 | 21.7 |
| Hospitalized COVID-19 | rs315666 | A | G | 0.728 | -0.185 | 0.039 | 1.61E-06 | 23 |
| Hospitalized COVID-19 | rs4853613 | C | T | 0.824 | -0.205 | 0.044 | 3.42E-06 | 21.6 |
| Hospitalized COVID-19 | rs72812493 | C | A | 0.01 | 0.872 | 0.18 | 1.29E-06 | 23.4 |
| Hospitalized COVID-19 | rs73974486 | G | A | 0.225 | -0.198 | 0.042 | 2.34E-06 | 22.3 |
| Severe COVID-19 | rs10514025 | C | A | 0.35 | -0.322 | 0.068 | 2.06E-06 | 22.5 |
| Severe COVID-19 | rs11044248 | A | G | 0.029 | 0.944 | 0.205 | 4.08E-06 | 21.2 |
| Severe COVID-19 | rs12059931 | A | T | 0.08 | 0.584 | 0.117 | 5.54E-07 | 25.1 |
| Severe COVID-19 | rs12244041 | C | T | 0.437 | -0.3 | 0.063 | 2.26E-06 | 22.4 |
| Severe COVID-19 | rs180913314 | G | A | 0.047 | 0.905 | 0.186 | 1.17E-06 | 23.6 |
| Severe COVID-19 | rs35727104 | C | T | 0.022 | 0.899 | 0.191 | 2.39E-06 | 22.2 |
| Severe COVID-19 | rs392108 | T | C | 0.777 | 0.398 | 0.078 | 3.55E-07 | 25.9 |
| Severe COVID-19 | rs55826734 | C | A | 0.025 | 1.126 | 0.228 | 7.65E-07 | 24.4 |
| Severe COVID-19 | rs75026182 | T | C | 0.178 | 0.448 | 0.098 | 4.56E-06 | 21 |
| Severe COVID-19 | rs76383282 | T | G | 0.014 | 1.229 | 0.257 | 1.80E-06 | 22.8 |
| Severe COVID-19 | rs9367106 | C | G | 0.317 | 0.357 | 0.068 | 1.42E-07 | 27.7 |

EAF, effect allele frequency;

No SNPs were removed because of relation to hearing loss according to PhenoScanner database. (PhenoScanner database: http://www.phenoscanner.medschl.cam.ac.uk).

The genetic instrumental variables were filtered with genome‐wide significance through *P<5e-06*, and removed Linkage disequilibrium (LD) using a clumping r2 cutoff of 0.001 within a 10 Mb window, using the 1000 Genomes Project Phase 3 (EAS) as the reference panel.

a: the F-statistic for each SNP was calculated as follows: F=(BATA/SE)^2

**Supplementary Table 8.** The results of pleiotropy test, Cochrane’s Q between COVID-19 and hearing loss (East Asian population)

| Exposure&Outcomes | Egger_intercept | *P* for pleiotropy^a^ | Cochrane’s Q | *P* for Cochrane’s Q^b^ |
| --- | --- | --- | --- | --- |
| SARS-CoV-2 infection | -0.059 | 0.22 | 8.08 | 0.43 |
| Hospitalized COVID-19 | -0.0033 | 0.92 | 9.73 | 0.28 |
| Severe COVID-19 | 0.0052 | 0.87 | 12.33 | 0.26 |

a: P-values for pleiotropy were derived from MR-Egger test and *P*-value<0.05 indicates a possible pleiotropic effect.

b: P-values for Cochrane’s Q were derived from Cochrane’s Q test and *P*-value<0.05 indicates a possible heterogeneity.

**Supplementary Table 9.** MR-PRESSO analysis for the association between COVID-19 and hearing loss(East Asian population).

| **Exposure** | **MR Analysis** | **Causal Estimate** | **Sd** | **T-stat** | **P-value** | **RSS_obs_** | **globalPvalue** | **outlier SNP** |
| --- | --- | --- | --- | --- | --- | --- | --- | --- |
| SARS-CoV-2 infection | Raw | -0.007213713 | 0.05495551 | -0.1312646 | 0.898807 | 10.13433 | 0.466 | 0 |
| Hospitalized COVID-19 | Raw | -0.04417521 | 0.05172131 | -0.8541008 | 0.4178891 | 12.35756 | 0.31 | 0 |
| Severe COVID-19 | Raw | -0.004295249 | 0.02715238 | -0.1581905 | 0.8774555 | 14.88784 | 0.26 | 0 |

**Supplementary Table 10.** The leave-one-out analysis for COVID-19 on hearing loss (East Asian population)

| Exposure | SNP | BETA | SE | *P*-value |
| --- | --- | --- | --- | --- |
| SARS-CoV-2 infection | rs10181451 | -0.0169 | 0.062 | 0.786 |
|  | rs12246187 | -0.0046 | 0.061 | 0.94 |
|  | rs12425722 | -0.0392 | 0.059 | 0.505 |
|  | rs12540473 | -5.00E-04 | 0.063 | 0.993 |
|  | rs12960174 | 0.0168 | 0.058 | 0.771 |
|  | rs183690023 | -0.0246 | 0.057 | 0.665 |
|  | rs315664 | 0.0266 | 0.058 | 0.644 |
|  | rs76497367 | -0.0143 | 0.061 | 0.814 |
|  | rs9367106 | -0.009 | 0.063 | 0.887 |
|  | **All** | **-0.0072** | **0.055** | **0.896** |
| Hospitalized COVID-19 | rs11066150 | -0.0497 | 0.057 | 0.384 |
|  | rs111553899 | -0.0328 | 0.057 | 0.567 |
|  | rs11919142 | -0.0329 | 0.056 | 0.559 |
|  | rs12175265 | -0.03 | 0.06 | 0.619 |
|  | rs144808322 | -0.0295 | 0.056 | 0.601 |
|  | rs315666 | -0.0257 | 0.054 | 0.631 |
|  | rs4853613 | -0.0706 | 0.049 | 0.151 |
|  | rs72812493 | -0.0806 | 0.05 | 0.11 |
|  | rs73974486 | -0.045 | 0.058 | 0.438 |
|  | **All** | **-0.0442** | **0.052** | **0.393** |
| Severe COVID-19 | rs10514025 | 0.0074 | 0.027 | 0.786 |
|  | rs11044248 | -0.0112 | 0.029 | 0.698 |
|  | rs12059931 | -0.0066 | 0.03 | 0.828 |
|  | rs12244041 | -0.0225 | 0.026 | 0.379 |
|  | rs180913314 | -0.0148 | 0.029 | 0.605 |
|  | rs35727104 | 2.00E-04 | 0.029 | 0.995 |
|  | rs392108 | 0.002 | 0.029 | 0.946 |
|  | rs55826734 | -0.0033 | 0.03 | 0.913 |
|  | rs75026182 | 0.0034 | 0.029 | 0.907 |
|  | rs76383282 | 0.0027 | 0.028 | 0.923 |
|  | rs9367106 | -0.0049 | 0.03 | 0.869 |
|  | **All** | **-0.0043** | **0.027** | **0.874** |
